# A first-in-human, randomized, controlled, subject- and reviewer-blinded multicenter study of Actamax™ Adhesion Barrier

**DOI:** 10.1007/s00404-016-4211-x

**Published:** 2016-11-14

**Authors:** Geoffrey H. Trew, George A. Pistofidis, Sara Y. Brucker, Bernhard Krämer, Nicole M. Ziegler, Matthias Korell, Henning Ritter, Alex McConnachie, Ian Ford, Alison M. Crowe, Trudy D. Estridge, Michael P. Diamond, Rudy L. De Wilde

**Affiliations:** 1 Consultant in Reproductive Medicine and Surgery, Hammersmith Hospital, Du Cane Road, London, W12 0HS UK; 2 Department of Gynecological Endoscopic Surgery, Lefkos Stavros Hospital, Athens, 115 28 Greece; 3 Department of Gynecology and Obstetrics, University of Tübingen, Tübingen, 72076 Germany; 4 Department of Obstetrics, Gynecology, and Gynecology Oncology, Pius Hospital, Oldenburg, 26121 Germany; 5 Department of Obstetrics and Gynecology, Johanna Etienne Krankenhaus, Neuss, 41462 Germany; 6 Robertson Centre for Biostatistics, University of Glasgow, Glasgow, G12 8QQ UK; 7 Corvus Communications Ltd, East Sussex, TN22 4PB UK; 8 Actamax Surgical Materials LLC, Wilmington, DE 19803 USA; 9 Department of Obstetrics and Gynecology, Augusta University, Augusta, GA 30912 USA

**Keywords:** Post-surgical adhesions, Hydrogel adhesion barrier, Gynecological laparoscopic surgery, Second-look laparoscopy, Adhesion barrier study

## Abstract

**Purpose:**

Post-surgical adhesions remain a significant concern following abdominopelvic surgery. This study was to assess safety, manageability and explore preliminary efficacy of applying a degradable hydrogel adhesion barrier to areas of surgical trauma following gynecologic laparoscopic abdominopelvic surgery.

**Methods:**

This first-in-human, prospective, randomized, multicenter, subject- and reviewer-blinded clinical study was conducted in 78 premenopausal women (18–46 years) wishing to maintain fertility and undergoing gynecologic laparoscopic abdominopelvic surgery with planned clinically indicated second-look laparoscopy (SLL) at 4–12 weeks. The first two patients of each surgeon received hydrogel, up to 30 mL sprayed over all sites of surgical trauma, and were assessed for safety and application only (*n* = 12). Subsequent subjects (*n* = 66) were randomized 1:1 to receive either hydrogel (Treatment, *n* = 35) or not (Control, *n* = 31); 63 completed the SLL.

**Results:**

No adverse event was assessed as serious, or possibly device related. None was severe or fatal. Adverse events were reported for 17 treated subjects (17/47, 36.2%) and 13 Controls (13/31, 41.9%). For 95.7% of treated subjects, surgeons found the device “easy” or “very easy” to use; in 54.5%, some residual material was evident at SLL. For 63 randomized subjects who completed the SLL, adjusted between-group difference in the change from baseline adhesion score demonstrated a 41.4% reduction for Treatment compared with Controls (*p* = 0.017), with a 49.5% reduction (*p* = 0.008) among myomectomy subjects (*n* = 34).

**Conclusion:**

Spray application of a degradable hydrogel adhesion barrier during gynecologic laparoscopic abdominopelvic surgery was performed easily and safely, without evidence of clinically significant adverse outcomes. Data suggest the hydrogel was effective in reducing postoperative adhesion development, particularly following myomectomy.

**Electronic supplementary material:**

The online version of this article (doi:10.1007/s00404-016-4211-x) contains supplementary material, which is available to authorized users.

## Introduction

Postoperative adhesions represent a major health care burden. They are the most frequent complication of abdominopelvic surgery, and the cause of significant patient morbidity including small bowel obstruction, infertility, chronic abdominal pain, and prolonged and complicated subsequent surgeries [1–4]. Gynecological abdominopelvic surgeries, including uterine myomectomy, ovarian cystectomy, lysis of adhesions and treatment of endometriosis, are particularly adhesiogenic [3, 5, 6]. While there have been considerable advances in surgical techniques including minimally invasive surgery, the risk of adhesions and related complications remains high, and for most therapeutic gynecological laparoscopic procedures, the comparative risk is similar to gynecological laparotomy [5] with approximately 20–40% of all infertility resulting from adhesions that involve the Fallopian tubes and ovaries [7–10].

In women wanting to preserve fertility, invasive procedures of this nature may not only severely impact fecundity, but they also put the patient at risk of other adhesion-related complications, including complications during any future abdominopelvic surgery [11–14]. Patients undergoing removal of a posterior fibroid are at great risk of adhesion formation, with high rates of adhesion formation to the posterior uterus, rectosigmoid and adnexa [15–20]. Because of the high risk of adhesions and the negative impact they have on clinical outcomes, many gynecological surgeons will recommend a second-look laparoscopy (SLL) a short time following the initial surgery, particularly in patients undergoing fertility-related surgery. This intervention provides the patient the opportunity to have a surgeon assess and potentially treat de novo and reformed adhesions or indeed other pathology resulting from the prior surgical procedure. It also allows the surgeon to assess the possibility of the patient conceiving without assistance and carrying an infant to full term [6, 21, 22]. Lysis of adhesions at an early stage in their formation is easier as they are more likely to be filmy and avascular, and it appears more successful than if undertaken later [23–25]. This may improve clinical outcomes, improve fertility [21, 26–28] and reduce other adhesion-related complications including the long-term risk of SBO and future re-operative complications. In women with endometriosis treated during surgery, a second-look procedure allows evaluation and treatment of any early recurrence as part of a protocol maximizing both cure rate and pregnancy outcomes [29]. A second-look laparoscopy following a myomectomy also allows the surgeon to examine the integrity of the uterine scar, in order to counsel the patient regarding attempting pregnancy [22, 30–32]. In subjects with extensive adhesion development, the subject may be advised to go directly to in vitro fertilization, adoption or surrogacy.

The risk versus benefit of a second-look laparoscopy is known. Adhesiolysis surgery in young, healthy women wishing to retain their fertility is beneficial, but economically many health and or insurers systems do not reimburse early second-look laparoscopy [28]. Undertaking a second-look laparoscopy within the study protocol in subjects where it is considered of clinical benefit, not only enables close evaluation of the abdominopelvic cavity, but also provides the opportunity for assessment of the potential therapeutic value of the study investigational device.

The development of post-surgical adhesions is multifactorial. History of previous surgeries, length of surgery, blood loss, suturing, surgical technique, length and location of uterine incision, and the size, number and location of myomas have all been shown to contribute to adhesion development following myomectomy [16, 17, 19, 30, 33]. Over the past 10 years, gynecological experts have collaborated to produce proposals for national and international guidance on adhesions and to assess the risk of adhesions [1, 2, 20, 33–39]. As part of a comprehensive adhesion reduction strategy using the basic tenets of microsurgery, all recommend consideration of anti-adhesion agents/barriers particularly in surgical procedures at high risk for adhesion development.

Actamax™ Adhesion Barrier (Actamax Surgical Materials LLC, Wilmington, DE) is a new investigational adhesion barrier device that is formed by mixing two aqueous solutions, dextran aldehyde and polyethylene glycol amine polymers, one of which contains a blue colorant. When sprayed together, the components form a thin, formed-in-place, tissue-adherent degradable hydrogel that directly overlies surgically traumatized tissue. This temporary hydrogel barrier allows damaged and apposing tissue surfaces the opportunity to heal without becoming abnormally attached during the immediate postoperative peritoneal healing period (3–5 days) [2, 40] when adhesions are most likely to form.

This first-in-human feasibility study was conducted primarily to assess the safety and manageability of the hydrogel adhesion barrier (“hydrogel”) and its application in a clinical setting. It was conducted in women wishing to retain their fertility, who were undergoing gynecologic laparoscopic surgery—including treatment of uterine fibroids, ovarian cysts, endometriosis and adhesions—followed by a clinically indicated SLL. Undertaking an SLL enables close evaluation of safety within the abdominopelvic cavity and provides the opportunity for assessment of the potential therapeutic value of the investigational device to be used in planning future studies [33]. From the patient’s perspective, the SLL serves as a valuable tool for their surgeon to formulate a likely prognosis on natural conception or need for assisted reproduction, as well as to lyse adhesions and treat recurring pathology at an early stage following the initial surgery [21, 22, 26, 28, 29].

## Materials and methods

### Trial design

This was a first-in-human, prospective, randomized (1:1), multicenter, controlled (surgery only), subject- and reviewer-blinded study. It was conducted between November 2013 and June 2014.

### Participants

Premenopausal women, aged 18–46 years, who wished to maintain their fertility and were undergoing gynecologic laparoscopic abdominopelvic surgery, with a planned clinically indicated SLL at 4–12 weeks (16-week upper limit), were eligible to be enrolled. Subjects who met all selection criteria were assigned intraoperatively to 1 of 2 substudies (myomectomy or other gynecologic pathology), and 1 of 2 arms of the myomectomy substudy (pure or hybrid), based on the major component of their surgery. All subjects provided written informed consent prior to undergoing any protocol-related procedures.

### Setting and ethical conduct

This study was conducted at centers in Germany (3) and Greece (1); all were tertiary referral centers for complex gynecologic laparoscopy. A total of 6 operating surgeons participated. The study, which was approved by the Independent Ethics Committees and relevant national regulatory authorities of each investigational site, was registered with ClinicalTrials.gov (NCT02260115).

### Procedures

The first two “initial usage” patients of each operating surgeon were assigned to receive hydrogel following their surgery and were assessed for its application and safety only. Subsequent patients underwent randomization once their primary surgery was complete and eligibility confirmed, including that an SLL was clinically indicated, before removal of the laparoscope. Subjects within each substudy and arm were randomized 1:1 to receive either hydrogel, up to 30 mL sprayed over all sites of surgical trauma (Treatment) or surgery only (Control). Follow-up assessments were conducted at hospital discharge, at 12–16 days, and at the SLL (or at 30–60 days if no SLL was undertaken). In cases where no SLL was performed, subjects were assessed for safety only. Both the primary surgery and SLL were video-recorded to allow for evaluation of hydrogel application and a separate blinded reviewer evaluation of adhesions and other pathologies throughout the abdominopelvic cavity.

### Interventions

For initial usage subjects and those randomized to Treatment, up to 30 mL of hydrogel (Actamax Surgical Materials LLC, Wilmington, DE) was applied laparoscopically to areas of the pelvis undergoing direct surgical trauma. For those in the myomectomy substudy, hydrogel was to be sprayed over the entire surface of the uterus and other areas of surgical trauma. All sites of surgical trauma were to be completely covered, allowing a margin of at least 3 cm around the traumatized site. The hydrogel was applied using a dual cannula CO_2_ gas-assisted endoscopic 5-mm applicator which mixed the two prefilled 5-mL syringes: one filled with an aqueous polymer solution of dextran aldehyde and FD&C Blue #1 (blue in color); the other filled with an aqueous polymer solution of two polyethylene glycol amines (clear in color). The syringe set, which was attached to a laparoscopic spray applicator and pressure regulator, was designed for use with the operating room carbon dioxide source. Subjects randomized to the Control underwent the primary gynecologic laparoscopic surgery without any further protocol-specified intervention.

### Outcomes

The primary study outcomes were safety related. These included the incidence of adverse events, and any abnormal vital signs or laboratory measures considered clinically significant by the operating surgeon. Secondary safety outcomes included surgeon experience with hydrogel application, presence of any residual material at SLL, postoperative pain, duration of hospital stay, and port site healing. Hydrogel application was assessed using a 5-point scale ranging from 1 = ‘‘very difficult” to 5 = ‘‘very easy”. Operating surgeons rated their overall satisfaction, viewing and handling of the device as well as various aspects of functionality. Based on these ratings, a mean ease of use score was also calculated. In the case of residual material, the operating surgeon recorded the qualitative amount and location of any material evident at the SLL. The blinded reviewer noted whether the considered material was present in the SLL videos and the study sponsor undertook an unblinded review of these same videos to estimate the volume of material based on comparisons with the known dimensions of the laparoscopic instruments present in the video. Efficacy outcomes were considered secondary. These included the formation of adhesions evident at the SLL, at each of 16 pre-specified anatomical sites and 5 regions (Supplemental Online Table 1). Adhesions were summarized by incidence, severity, extent and adhesion score. Adhesion efficacy outcomes were based on the change from baseline at SLL in the adhesion score, calculated as the combined score of the severity and extent of adhesions at that site or region.

### Sample size

It was prospectively estimated that 75 enrolled subjects were needed so that at least 60 subjects, randomized into equal numbers to receive surgery with or without hydrogel, underwent the SLL. Of the 75, it was expected that less than 5% (approximately 3 subjects) would not complete the study and that, with 6 operating surgeons, 12 subjects would serve as initial usage subjects. For an adverse outcome that occurs in 5% of treated subjects, this sample ensured that there was >90% power to observe an event in at least one of the 42 treated subjects (12 initial usage plus 30 randomized).

### Randomization

Randomization was stratified by surgeon and by surgical subgroup. Randomization was conducted in permuted blocks of size two within each stratum, to ensure approximate treatment balance within each surgeon/subgroup at the end of the study. That is, for each surgeon, every two consecutive randomized subjects within each subgroup were allocated 1:1 between Treatment and Control. The block size was intentionally small to accommodate the small number of subjects recruited by each surgeon for this first-in-human study. Allocations were obtained by telephone to an interactive voice response system or via the electronic case report form managed by the Robertson Centre for Biostatistics, University of Glasgow, so that allocations were concealed from study sites until the time of randomization; clinical sites were not informed of the permuted block size.

### Blinding

Study subjects remained blinded to their treatment allocation beyond study completion. Of necessity, the operating surgeon was not blinded. For all subjects who received hydrogel, the application video segment from the primary surgery was removed and sent to an evaluator assigned to assess the quality of hydrogel application. Following the SLL, copies of paired videos for the primary surgery (with application section removed) and SLL were provided to the blinded evaluator assigned to assess adhesions.

### Statistical methods

Data were summarized for baseline characteristics, the primary surgery including hydrogel application, and safety and efficacy outcomes. Adverse event data were tabulated. Categorical data were compared between randomized groups using Fisher’s exact test; continuous data were compared between randomized groups using the Wilcoxon–Mann–Whitney test. For efficacy analyses, changes in adhesion scores were compared between randomized groups using linear regression models, adjusted for substudy and baseline adhesions scores, and reported as the estimated treatment effect difference with a 95% confidence interval and *p* value. Analyses were carried out using *R* for Windows v3.0.0 and SAS for Windows v9.2.

## Results

### Participants

**Fig. 1:**
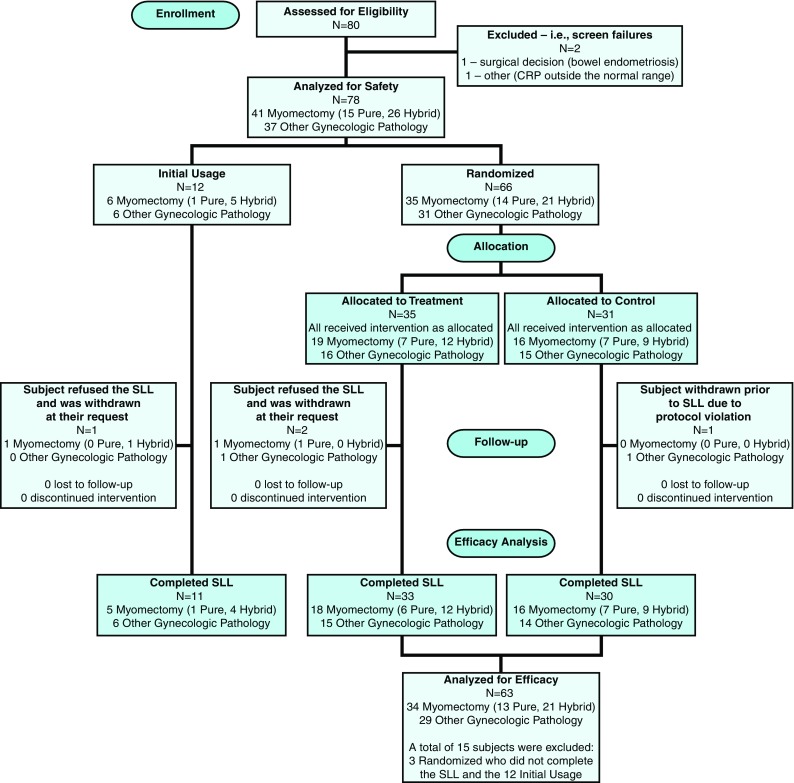
CONSORT flow diagram

The flow of study subjects from initial screening to study completion is illustrated in Fig. 1. A total of 80 patients were screened for enrollment; two were screen failures. The remaining 78 subjects formed the Safety Population. Approximately half (41, 52.6%) were allocated to the myomectomy substudy. The 63 randomized patients who completed the SLL formed the Efficacy population (33 Treatment; 30 Control). In total, 74 subjects (74/78, 95%) including 11 initial usage subjects, underwent the SLL and completed the study; 4 enrolled subjects (5%) refused the SLL and were included in the Safety Population.

### Baseline characteristics

The 78 study subjects had a mean age of 33.6 years (Supplemental Online Table 2); almost all (76/78, 97.4%) were racially identified as “white.” Their mean weight and BMI were 68.9 kg and 24.8 kg/m^2^, respectively. Among randomized subjects, more Treatment subjects were smokers (16/35, 45.7% vs. 5/31 Control, 16.1%); fewer Treatment subjects had a history of treatment for uterine fibroids (2/35, 5.7% vs. 7/31 Control, 22.6%). Otherwise, differences between groups and substudies in baseline characteristics were unremarkable. All baseline vital signs were assessed as of no clinical concern, and no baseline laboratory measure that was outside the normal range was judged as clinically significant by the treating surgeon.

### Primary gynecological laparoscopy record

The mean duration of the primary surgery was 90.8 ± 48.7 min (Table 1). Fifty-one subjects (51/78, 65.4%) underwent surgical removal of fibroids, with no difference in the percentages of Treatment and Control subjects undergoing this procedure (62.9% vs. 74.2%, respectively, *p* = 0.429). For the 72 subjects (72/78, 92.3%) whose primary surgery videos underwent independent review, 52 (52/72, 72.2%) had at least one adhesion; the mean number of anatomical sites with baseline adhesions was 3.1 ± 3.1. Despite randomization, statistically significantly more subjects in the Treatment group had baseline adhesions: 29/33, 87.9% vs. 18/30 Control, 60.0%, *p* = 0.019. Treatment subjects also had more anatomical sites with baseline adhesions, and a greater maximum severity, mean extent, and mean adhesion score. Among substudies, differences between Treatment and Controls were particularly marked in other gynecologic pathology subjects.

**Table 1:** Primary surgery record—surgical procedures and summary of adhesions for the entire abdominal cavity

	All (*N* = 78)	Initial usage (*N* = 12)	Randomized (*N* = 66)		
			Treatment (*N* = 35)	Control (*N* = 31)	*p* value
*Surgical procedure/characteristic*					
Myomectomy^a^, *n* (%)	51 (65.4%)	6 (50.0%)	22 (62.9%)	23 (74.2%)	0.429^e^
No. of fibroids removed, mean ± SD [min, max]	3.0 ± 2.1 [1, 10]	2.8 ± 1.9 [1, 6]	2.9 ± 2.0 [1, 8]	3.1 ± 2.3 [1, 10]	0.824^f^
Total wt. of fibroids (g), mean ± SD [min, max]	77.3 ± 69.2 [3, 300]	98.8 ± 103.0 [8, 300]	64.4 ± 56.8 [7, 226]	83.9 ± 71.0 [3, 232]	0.625^f^
Surgery for ovarian cysts, *n* (%)	14 (17.9%)	4 (33.3%)	7 (20.0%)	3 (9.7%)	0.314^e^
No. of cysts removed, mean ± SD [min, max]	1.3 ± 0.5 [1, 2]	1.5 ± 0.7 [1, 2]	1.4 ± 0.6 [1, 2]	1.0 ± 0.0 [1, 1]	0.324^f^
Adhesiolysis, *n* (%)	48 (61.5%)	4 (33.3%)	27 (77.1%)	17 (54.8%)	0.070^e^
Surgery for endometriosis, *n* (%)	40 (51.3%)	5 (41.7%)	17 (48.6%)	18 (58.1%)	0.469^e^
Diagnostic hysteroscopy, *n* (%)	45 (57.7%)	6 (50.0%)	21 (60.0%)	18 (58.1%)	1.000^e^
Other gynecologic procedure, *n* (%)	1 (1.3%)	1 (8.3%)	0 (0.0%)	0 (0.0%)	1.000^e^
Duration of surgery (min), mean ± SD [min, max]	90.8 ± 48.7 [16, 229]	91.1 ± 37.3 [35, 156]	91.7 ± 44.9 [43, 207]	89.8 ± 57.3 [16, 229]	0.537^f^
*Adhesion parameter at initial surgery*					
Any adhesions present, *n* (% of those with ≥1 assessable site)	52/72 (72.2%)	5/9 (55.6%)	29/33 (87.9%)	18/30 (60.0%)	0.019^e^
No. of sites with adhesions, mean ± SD [min, max]	3.1 ± 3.1 [0, 11]	2.2 ± 2.7 [0, 7]	4.2 ± 3.4 [0, 11]	2.3 ± 2.6 [0, 10]	0.016^f^
Maximum severity score^b^, mean ± SD					
All (*N* = 72) Pure myomectomy (*n* = 14) Hybrid myomectomy (*n* = 24) Other gynecologic pathology (*n* = 34)	1.42 ± 1.06 0.36 ± 0.74 1.29 ± 0.91 1.94 ± 0.92	1.22 ± 1.20 – 0.67 ± 1.15 1.80 ± 1.10	1.82 ± 0.95 0.83 ± 0.98 1.58 ± 0.79 2.40 ± 0.63	1.03 ± 1.00 0.00 ± 0.00 1.11 ± 0.93 1.50 ± 0.94	0.003^f^ 0.053^f^ 0.284^f^ 0.009^f^
Mean extent score^c^, mean ± SD					
All (*N* = 72) Pure myomectomy (*n* = 14) Hybrid myomectomy (*n* = 24) Other gynecologic pathology (*n* = 34)	0.25 ± 0.32 0.03 ± 0.05 0.19 ± 0.26 0.38 ± 0.37	0.23 ± 0.34 – 0.04 ± 0.07 0.39 ± 0.39	0.32 ± 0.36 0.06 ± 0.07 0.19 ± 0.09 0.52 ± 0.44	0.18 ± 0.27 0.00 ± 0.00 0.25 ± 0.41 0.22 ± 0.20	0.020^f^ 0.051^f^ 0.446^f^ 0.027^f^
Adhesion score^d^, mean ± SD					
All (*N* = 72) Pure myomectomy (n = 14) Hybrid myomectomy (n = 24) Other gynecologic pathology (*n* = 34)	1.67 ± 1.29 0.38 ± 0.80 1.48 ± 1.04 2.32 ± 1.19	1.45 ± 1.44 – 0.71 ± 1.23 2.19 ± 1.33	2.14 ± 1.19 0.90 ± 1.05 1.77 ± 0.82 2.92 ± 0.98	1.21 ± 1.19 0.00 ± 0.00 1.36 ± 1.19 1.72 ± 1.09	0.004^f^ 0.053^f^ 0.352^f^ 0.006^f^

^a^Only 41 of the 51 subjects who underwent a myomectomy procedure during their primary surgery were assigned to the myomectomy substudy based on myomectomy being the major component of that surgery

^b^Severity was assessed on 4-point scale: 0 = no adhesions; 1 = filmy/no vascularity; 2 = dense/vascular; 3 = cohesive (i.e., two surfaces directly adhered with no clear bands)

^c^Extent was also assessed on a 4-point scale: 0 = none; 1 = ≤1/3 area of anatomical site; 2 = 1/3–2/3 area of anatomical site; 3 = ≥2/3 area of anatomical site

^d^Adhesion score = maximum severity + mean extent scores at all assessable sites

^e^Fisher’s exact test *p* values

^f^Wilcoxon–Mann–Whitney test *p* values

### Adverse events

Thirty subjects (30/78, 38.5%) experienced a total of 43 adverse events, including 2 events in 2 initial usage subjects (2/12, 16.7%) (Table 2). The proportion of randomized subjects who experienced at least one event was no different between groups: 24 events were reported for 15 Treatment subjects (15/35, 42.9%); 17 events were reported for 13 Controls (13/31, 41.9%). In total, 26 events were reported for 17 hydrogel-treated subjects (2/12 initial usage + 15/35 Treatment, 17/47, 36.2%). No event was assessed as serious, or as possibly/probably related to the investigational device or to any study procedures. None was fatal or assessed as severe. Five events were deemed either highly probably or probably related to another device or agent used: 1 case of metrorrhagia, dysmenorrhea and abdominal pain, 1 of suture granuloma, and 1 of phlebitis (all Treatment subjects); 2 cases of hypersensitivity reaction to sutures (1 Treatment, 1 Control). The most common adverse events, occurring in 5% or more of subjects (*n* = 4), were “infections and infestations” reported for seven patients (Table 2). These included influenza, nasopharyngitis, urinary tract infection and viral infection. The second most common events were “nervous system disorders” reported for six patients, which included headache, dizziness, and paresthesia. There were no differences in the percentages of randomized Treatment and Control subjects who experienced events in either of these classes.

**Table 2:** Adverse events

	All (*N* = 78)	Initial usage (*N* = 12)	Randomized (*N* = 66)	
			Treatment (*N* = 35)	Control (*N* = 31)
Number of patients who had an AE	30 (38.5%)	2 (16.7%)	15 (42.9%)	13 (41.9%)
Number of adverse events	43	2	24	17
Adverse device effect	0	0	0	0
Serious adverse event	0	0	0	0
Relationship with investigational device,* n* (%)^a^				
Not related Unlikely to be related Possibly/probably related	37 (86.0%) 6 (14.0%) 0	2 (100%) 0 0	19 (79.2%) 5 (20.8%) 0	16 (94.1%) 1 (5.9%) 0
Relationship with study procedure,* n* (%)^a^				
Not related Unlikely to be related Possibly/probably related	37 (86.0%) 6 (14.0%) 0	2 (100%) 0 0	19 (79.2%) 5 (20.8%) 0	16 (94.1%) 1 (5.9%) 0
Relationship with other device/agent used,* n* (%)^a^				
Not related Unlikely to be related Possibly related Probably/highly related	32 (74.4%) 6 (14.0%) 0 5 (11.6%)	2 (100%) 0 0 0	15 (62.5%) 5 (20.8%) 0 4 (16.7%)	15 (88.2%) 1 (5.9%) 0 1 (5.9%)
Severity,* n* (%)^a^				
Mild Moderate Severe	28 (65.1%) 15 (34.9%) 0	1 (50%) 1 (50%) 0	15 (62.5%) 9 (37.5%) 0	12 (70.6%) 5 (29.4%) 0
*Commonly occurring events* (≥*5% of subjects*)* by system organ class and preferred term* ^b^				
Infections and infestations Influenza Nasopharyngitis Urinary tract infection Viral infection	7 (9.0%) 2 (2.6%) 2 (2.6%) 2 (2.6%) 1 (1.3%)	0	4 (11.4%) 2 1 0 1	3 (9.7%) 0 1 2 0
Nervous system disorders Dizziness Headache Paresthesia	6 (7.7%) 1 (1.3%) 4 (5.1%) 1 (1.3%)	0	3 (8.6%) 0 2 1	3 (9.7%) 1 2 0
Gastrointestinal disorders Abdominal pain Constipation Vomiting	4 (5.1%) 2 (2.6%) 1 (1.3%) 1 (1.3%)	0	1 (2.9%) 1 0 0	3 (9.7%) 1 1 1
Immune system disorders Drug hypersensitivity Hypersensitivity Seasonal allergy	4 (5.1%) 1 (1.3%) 2 (2.6%) 1 (1.3%)	0	2 (5.7%) 1 1 0	2 (6.5%) 0 1 1
Reproductive system and breast disorders Dysmenorrhea + metrorrhagia Ovarian cyst Uterine disorder	4 (5.1%) 1 (1.3%) 2 (2.6%) 1 (1.3%)	0	3 (8.6%) 1 1 1	1 (3.2%) 0 1 0
Skin and subcutaneous tissue disorders Erythema + pruritus Rash Rash pruritic	4 (5.1%) 1 (1.3%) 2 (2.6%) 1 (1.3%)	0	2 (5.7%) 0 2 0	2 (6.5%) 1 0 1

^a^As determined by operating surgeon, displayed as a percent of events

^b^As determined by operating surgeon, displayed as a percent of subjects

### Vital signs and clinical laboratory measures

As at baseline, no vital sign measures at the SLL were assessed as abnormal or of clinical concern. Most subjects had at least one laboratory measurement outside the normal range at either the discharge visit (75/78, 96.2%) or SLL (66/78, 84.6%); no abnormal measure was judged by the treating surgeon to be of clinical significance.

### Hydrogel application

Hydrogel was applied in 47 subjects: 12 initial usage and 35 randomized to Treatment. A mean of 11.3 ± 4.3 mL was applied (range of 4–20 mL); application was complete in a mean of 6.2 ± 2.8 min (range of 1–15 min). A single syringe set (10 mL of product) was used for 33 of the 47 subjects (70.2%); two syringe sets were used for the other 14 (29.8%). Operating surgeons were satisfied with hydrogel manageability, assigning a rank of “easy” or “very easy” for each of overall satisfaction, viewing and handling for 45 of the 47 subjects (95.7%) in whom it was applied (Supplemental Fig. 1). Where 12 specific aspects of functionality were assessed, the mean ease of use score was 4.4 (1 = ‘‘very difficult”, 5 = ‘‘very easy”): 4.2 for initial usage subjects and 4.5 for Treatment subjects (Wilcoxon–Mann–Whitney test *p* = 0.039, data not displayed).

### Residual material at SLL

A total of 44 subjects (11 initial usage, 33 Treatment) who received hydrogel following their primary surgery underwent the SLL. For 20 subjects (20/44, 45.5%), the blinded independent reviewer of the SLL videos found no evidence of residual material at any anatomical site. For the other 24 (24/44, 54.5%), residual material was observed at a mean of two sites (range of 0–12 sites). There was no evidence that the presence of residual material was associated with the substudy (*p* = 0.666), the volume of hydrogel applied (<10, =10, >10 mL; *p* = 0.646) or with the duration between the primary surgery and SLL (≤8, >8 and ≤10, >10 weeks; *p* = 0.692). Residual material was found more commonly at sites where hydrogel had been applied during the primary surgery. Based on the unblinded review of the SLL videos, the volume of residual material was estimated to be a mean of 1.0 mL, representing 9.1% of the mean volume applied during the primary surgery. There was no indication that any adverse outcomes were associated with the presence of residual material; no intra-abdominal or postoperative infections at the operative site were observed in any subject.

### Other safety outcomes

Pain was assessed as “greater than expected” for one Control subject at hospital discharge, and for three subjects (two Treatment, one Control) at SLL. No subject experienced abdominal pain that was deemed to be “of clinical concern”. Hospital stay was assessed as “longer than expected” in only two subjects (2.6%)—both Controls (neither was considered a serious adverse event). The primary surgery had been conducted using three ports in 29 subjects (29/78, 37.2%), four ports in 46 subjects (46/78, 59.0%) and five ports in 3 (3/78, 3.8%). At hospital discharge, port site healing was assessed as “uncomplicated” in 97.4% of subjects (35/35 Treatment, 100%; 28/30 Control, 93.3%) and as “complicated” for two Control subjects; no subject experienced port site healing that was assessed to be “of clinical concern” at either the time of discharge or the SLL.

### Efficacy

For the Efficacy Population (*n* = 63), from the initial surgery to SLL, Treatment subjects showed a mean reduction in adhesion score at sites of surgery throughout the abdominal cavity of 0.51 ± 1.62 points; in marked contrast, Control subjects showed an increase of 0.95 ± 1.89 points (Table 3). Based on linear regression analysis, adjusting for substudy and baseline adhesion score, the adhesion score in the Treatment group was estimated to be 0.96 (95% CI 0.18, 1.74) points lower than in the Control group (*p* = 0.017); this difference represented 41.4% of the mean adhesion score for Control patients. For myomectomy substudy subjects (*n* = 34), where the primary efficacy endpoint was limited to the posterior uterus site, the adjusted difference between groups represented a 38.2% reduction in adhesion score for Treatment subjects compared with Controls (*p* = 0.086). When the analysis for myomectomy substudy subjects was expanded to include all sites of surgery throughout the abdominal cavity, the adjusted difference between groups represented a statistically significant 49.5% reduction in adhesion score for Treatment subjects compared with Controls (*p* = 0.008). No statistically significant differences were observed for either of the primary efficacy outcomes assessed for Other Gynecological Pathology substudy subjects. Regardless of whether or not treated subjects had evidence of residual material present at SLL, scoring of adhesions by the independent video reviewer did not differ significantly for any of the efficacy outcomes assessed (Supplemental Online Tables 3 and 4).

**Table 3:** Efficacy analyses—adhesion scores at sites of surgery

Adhesion score^a^ by study population	Treatment	Control	*p* value
All efficacy subjects, *n*	33	30	
Adhesion score, abdominal cavity, mean ± SD			
Primary surgery	2.34 ± 1.45	1.36 ± 1.40	0.008^b^
SLL	1.83 ± 1.50	2.32 ± 1.48	0.228^b^
Change	– 0.51 ± 1.62	0.95 ± 1.89	0.002^b^
Unadjusted between-group difference	1.46		
Adjusted between-group difference (95% CI)^c^	0.96 (0.18, 1.74)		0.017
–as % of adhesion score for controls at SLL	↓ 41.4%		
Myomectomy substudy subjects (pure + hybrid), *n*	18	16	
Adhesion score, posterior uterus, mean ± SD			
Primary surgery	0.33 ± 0.77	0.19 ± 0.75	0.422^b^
SLL	1.61 ± 1.38	2.56 ± 1.71	0.058^b^
Change	1.28 ± 1.56	2.38 ± 1.82	0.068^b^
Unadjusted between-group difference	1.10		
Adjusted between-group difference (95 % CI)^c^	0.98 (−0.15, 2.10)		0.086
–as % of adhesion score for controls at SLL	↓ 38.2%		
Adhesion score, abdominal cavity, mean ± SD			
Primary surgery	1.52 ± 1.22	0.90 ± 1.30	0.097^b^
SLL	1.54 ± 1.32	2.74 ± 1.36	0.010^b^
Change	0.02 ± 1.65	1.85 ± 1.62	0.003^b^
Unadjusted between-group difference	1.83		
Adjusted between-group difference (95 % CI)^c^	1.36 (0.37, 2.34)		0.008
–as % of adhesion score for controls at SLL	↓ 49.5%		
Other gynecologic pathology substudy subjects, *n*	15^d^	14^d^	
Adhesion score, combined adnexa, mean ± SD			
Primary surgery	2.97 ± 1.67	3.10 ± 1.14	1.000^b^
SLL	2.81 ± 1.79	1.80 ± 1.96	0.342^b^
Change	−0.17 ± 2.21	−1.30 ± 1.72	0.401^b^
Unadjusted between-group difference	−1.13		
Adjusted between-group difference (95 % CI)^c^	−1.04 (−3.10, 1.02)		0.298
–as % of adhesion score for controls at SLL	↑ 57.9%		
Adhesion score, abdominal cavity, mean ± SD			
Primary surgery	3.32 ± 1.05	1.90 ± 1.35	0.009^b^
SLL	2.18 ± 1.67	1.83 ± 1.51	0.400^b^
Change	−1.14 ± 1.38	−0.07 ± 1.67	0.149^b^
Unadjusted between-group difference	1.07		
Adjusted between-group difference (95 % CI)^c^	0.46 (−0.86, 1.77)		0.482
–as % of adhesion score for controls at SLL	↓ 24.9%		

^a^Maximum severity + mean extent at sites of surgery with adhesions at the SLL for the specified region or site

^b^Between group comparisons based on Wilcoxon–Mann–Whitney tests

^c^Estimate based on linear regression model adjusting for substudy and baseline score

^d^Not all subjects had adnexal adhesions (*n* = 13 Treatment, 5 Control at the combined adnexa)

## Discussion

Usage data collected for the 47 subjects in whom hydrogel was administered, indicate that application during gynecologic laparoscopic surgery was neither difficult nor time consuming, and was performed safely without evidence of any serious adverse events or clinically significant adverse outcomes for any safety measure assessed.

As this was a first-in-human study, one key component was the evaluation of the surgeons’ ability to handle the device effectively. When “ease of use” assessments were converted to numerical scores for the 12 specific aspects of device functionality assessed, the mean score for initial usage subjects was statistically significantly lower than that of randomized Treatment subjects, suggesting that surgeons found the device easier to use with practice. Still, it is clear from the data displayed in Supplemental Online Fig. 1 that surgeons performed all aspects of application with a high degree of ease for almost all subjects. For example, in 89.4% of cases, surgeons reported that manipulation of the sprayer tip was either “easy” or “very easy”, with the remaining cases assessed as “okay”.

Our data suggest that in this first-in-human study, hydrogel application was of particular benefit in myomectomy patients. When evaluated for all subjects in the Efficacy Population as well as for those in the myomectomy substudy, statistically significant treatment-related reductions in the change from baseline adhesion scores of 41.4% and 49.5%, respectively, were observed in these populations (*p* = 0.017 and *p* = 0.008, respectively). These data indicate performance that is equal or better than in other studies, reported between 2003 and 2008, in which gel-based post-surgical adhesion barriers were applied [17, 41–47].

Limitations to this study include that, although efficacy analyses were adjusted for baseline mean adhesion scores, we cannot exclude the possibility that differences between groups in the number, severity and extent of adhesions at baseline influenced our conclusions. In addition, this 78-patient first-in-human study was not powered on the basis of detecting differences between randomized groups for efficacy outcomes. It is also recognized that while this study was designed with a >90% power to observe an adverse event that occurs in 5% of treated subjects, the power to detect the event in this small sample size has a 95% confidence interval of between 0 and 12%. It is our contention that these data are sufficiently promising that a larger clinical efficacy and safety study is warranted to corroborate these findings.

Another potential limitation of this study was that the assessment of efficacy outcomes might have been influenced by the presence of small amounts of residual material at the SLL. The independent “blinded” reviewer assessing postoperative adhesions on SLL videotape whilst being blind to the randomization allocation of the patient would believe, if they observed any residual material to be present, that a subject had received hydrogel during their primary surgery. To investigate the possibility of bias from such knowledge, the primary efficacy analyses were repeated, splitting the Treatment group into those where residual material was or was not observed. No evidence was found that observation of residual material affected the efficacy of the Actamax™ Adhesion Barrier as assessed by the independent reviewer’s scoring of adhesions.

Our observations suggest that a thin flexible tissue-adherent bioabsorbable hydrogel that can be laparoscopically applied, such as the Actamax™ Adhesion Barrier, will prove to be an important anti-adhesion surgical adjuvant to reduce postoperative adhesion development.

## Conclusion

In summary, in this first-in-human clinical study, spray application of a degradable polyethylene glycol-based hydrogel adhesion barrier during laparoscopic gynecologic surgery was performed easily, safely and without evidence of any clinically significant adverse outcomes. Our data also suggest that the hydrogel was effective in reducing adhesions, particularly following myomectomy. Further study is warranted to corroborate these promising initial results.

## Electronic supplementary material

Below is the link to the electronic supplementary material.

Supplementary material 1 (DOCX 82 kb)

Supplementary material 2 (DOCX 14 kb)

Supplementary material 3 (DOCX 14 kb)

Supplementary material 4 (DOCX 13 kb)

Supplementary material 5 (DOCX 13 kb)
