# Supplementary material for: A first-in-human, randomized, controlled, subject- and reviewer-blinded multicenter study of Actamax™ Adhesion Barrier
Source: Arch Gynecol Obstet. 2016 Nov 14;295(2):383–95. doi: 10.1007/s00404-016-4211-x (PMC5281664; doi:10.1007/s00404-016-4211-x)
Supplement: Supplementary file 2 — Supplementary material 2 (DOCX 14 kb) [file 404_2016_4211_MOESM2_ESM.docx]

**Supplemental Online Table 1. Sites, Regions and Scoring of Adhesions, and Efficacy Outcomes**

| **Anatomical sites (16) of adhesion assessment** | | |
| --- | --- | --- |
| 1. Anterior uterus  2. Posterior uterus  3. Right fallopian tube  4. Right ovary  5. Right pelvic sidewall  6. Left fallopian tube | 7. Left ovary  8. Left pelvic sidewall  9. Posterior cul-de-sac  10. Bladder  11. Anterior cul-de-sac | 12. Anterior abdominal wall  13. Umbilical port site  14. Small bowel  15. Omentum  16. Large bowel |
| **Anatomical regions (5) of adhesion assessment**   - Abdominal cavity – combination of all anatomical sites (sites 1-16) - Uterus only – combination of anterior and posterior uterus (sites 1 and 2) - Right adnexa – combination of right fallopian tube and right ovary (sites 3 and 4) - Left adnexa – combination of left fallopian tube and left ovary (sites 6 and 7) - Combined adnexa – combination of right and left adnexa (sites 3, 4, 6 and 7) | | |
| **Scoring of adhesions**   - Presence of adhesions, and whether this was a site of de novo or reformed adhesions - Severity: 0 = no adhesions; 1 = filmy/no vascularity; 2 = dense/vascular; 3 = cohesive - Extent: 0 = none; 1 = ≤ 1/3 area of site; 2 = 1/3 to 2/3 area of site; 3 = ≥ 2/3 area of site - Adhesion Score = severity + extent scores **per site** - Adhesion Score = maximum severity + mean extent scores **per region** | | |
| **Primary efficacy outcomes**   - **All subjects:** Change from baseline at SLL in the Adhesion Score at all sites of surgery – i.e., throughout the entire abdominal cavity - **Myomectomy substudy:** Change from baseline at SLL in the Adhesion Score at the posterior uterus - **Other Gynecologic Pathology substudy:** Changes from baseline at SLL in the Adhesion Scores at sites of surgery at the combined adnexa, and at all sites of surgery   **Additional efficacy outcome**   - **Myomectomy substudy:** Change from baseline at SLL in the Adhesion Score at all sites of surgery | | |
