# Supplementary material for: A first-in-human, randomized, controlled, subject- and reviewer-blinded multicenter study of Actamax™ Adhesion Barrier
Source: Arch Gynecol Obstet. 2016 Nov 14;295(2):383–95. doi: 10.1007/s00404-016-4211-x (PMC5281664; doi:10.1007/s00404-016-4211-x)
Supplement: Supplementary file 3 — Supplementary material 3 (DOCX 14 kb) [file 404_2016_4211_MOESM3_ESM.docx]

**Supplemental Online Table 2. Baseline Characteristics, including Demographics, Vital Signs and Gynecologic and Abdominopelvic History**

| **Characteristic / History ^a^** | **All**  **(N=78)** | **Initial Usage**  **(N=12)** | **Randomized (N=66)** | |
| --- | --- | --- | --- | --- |
|  |  |  | **Treatment (N=35)** | **Control (N=31)** |
| Age (years), mean ± SD | 33.6 ± 5.9 | 34.8 ± 7.2 | 32.7 ± 5.7 | 34.1 ± 5.5 |
| Height (cm), mean ± SD | 166.4 ± 5.9 | 166.4 ± 5.7 | 166.0 ± 5.4 | 166.8 ± 6.8 |
| Weight (kg), mean ± SD | 68.9 ± 17.0 | 66.4 ± 15.2 | 70.3 ± 20.3 | 68.3 ± 13.4 |
| BMI (kg/m^2^), mean ± SD | 24.8 ± 5.5 | 23.9 ± 5.2 | 25.4 ± 6.7 | 24.5 ± 4.2 |
| Any history of smoking, n (%) | 25 (32.1%) | 4 (33.3%) | 16 (45.7%) | 5 (16.1%) |
| Vital signs |  |  |  |  |
| Systolic BP (mmHg), mean ± SD | 123.8 ± 14.4 | 125.6 ± 15.1 | 123.1 ±15.6 | 124.0 ± 13.0 |
| Diastolic BP (mmHg), mean ± SD | 74.4 ±10.1 | 76.2 ± 7.2 | 73.9 ± 12.3 | 74.2 ± 8.4 |
| Heart rate (bpm), mean ± SD | 77.6 ± 8.7 | 77.0 ± 8.9 | 77.3 ± 10.2 | 78.1 ± 6.7 |
| Previous abdominopelvic surgery, n (%) |  |  |  |  |
| Gynecological | 26 (33.3%) | 3 (25.0%) | 12 (34.3%) | 11 (35.5%) |
| Non-gynecological – e.g., hernia | 18 (23.1%) | 3 (25.0%) | 8 (22.9%) | 7 (22.6%) |
| Treatment previously received for, n (%) |  |  |  |  |
| Uterine fibroids | 11 (14.1%) | 2 (16.7%) | 2 (5.7%) | 7 (22.6%) |
| Ovarian cysts | 9 (11.5%) | 3 (25.0%) | 3 (8.6%) | 3 (9.7%) |
| Adhesions | 5 (6.4%) | 2 (16.7%) | 2 (5.7%) | 1 (3.2%) |
| Endometriosis | 17 (21.8%) | 3 (25.0%) | 7 (20.0%) | 7 (22.6%) |
| Menorrhagia | 5 (6.4%) | 0 (0.0%) | 2 (5.7%) | 3 (9.7%) |
| Dysmenorrhoea | 11 (14.1%) | 1 (8.3%) | 5 (14.3%) | 5 (16.1%) |
| Other gynecological problem | 14 (17.9%) | 1 (8.3%) | 9 (25.7%) | 4 (12.9%) |

^a^ as recorded at the screening visit
