# Supplementary material for: A first-in-human, randomized, controlled, subject- and reviewer-blinded multicenter study of Actamax™ Adhesion Barrier
Source: Arch Gynecol Obstet. 2016 Nov 14;295(2):383–95. doi: 10.1007/s00404-016-4211-x (PMC5281664; doi:10.1007/s00404-016-4211-x)
Supplement: Supplementary file 4 — Supplementary material 4 (DOCX 13 kb) [file 404_2016_4211_MOESM4_ESM.docx]

**Supplemental Online Table 3. Bias Analysis – Efficacy Outcomes for the Entire Abdominal Cavity at SLL by Presence/Absence of Residual Material – Treated Subjects, Efficacy Population (N=33)**

| **Efficacy Outcome** | **Treatment (N=33)** | | | |  |
| --- | --- | --- | --- | --- | --- |
|  | **No Residue**  **Present (N=13) ^a^** | | **Residue**  **Present (N=20)** | |  |
|  | **No**  **Residue Seen (N=10) ^b^** | **Residue Seen**  **(N=3)** | **No**  **Residue Seen**  **(N=6)** | **Residue Seen (N=14)** | **Control**  **(N=30)** |
| Adhesion incidence, n (%) | 8 (80.0%) | 2 (66.7%) | 4 (66.7%) | 10 (71.4%) | 24 (80.0%) |
| Maximum severity, mean ± SD | 1.40 ± 1.07 | 1.33 ­± 1.15 | 1.50 ± 1.38 | 1.64 ± 1.22 | 1.80 ± 1.10 |
| Mean extent, mean ± SD | 0.27 ± 0.31 | 0.09 ± 0.08 | 0.30 ± 0.28 | 0.23 ± 0.18 | 0.24 ± 0.23 |
| Adhesion Score, mean ± SD | 1.67 ± 1.33 | 1.42 ± 1.23 | 1.80 ± 1.63 | 1.87 ± 1.36 | 2.04 ± 1.23 |

^a^ no residue present/residue present refer to whether residue was observed by the sponsor’s secondary video review;

^b^ no residue seen/residue seen refers to whether the independent reviewer observed material in that same subject
