# Supplementary material for: A first-in-human, randomized, controlled, subject- and reviewer-blinded multicenter study of Actamax™ Adhesion Barrier
Source: Arch Gynecol Obstet. 2016 Nov 14;295(2):383–95. doi: 10.1007/s00404-016-4211-x (PMC5281664; doi:10.1007/s00404-016-4211-x)
Supplement: Supplementary file 5 — Supplementary material 5 (DOCX 13 kb) [file 404_2016_4211_MOESM5_ESM.docx]

**Supplemental Online Table 4. Statistical Tests for Bias Analysis – Efficacy Outcomes for the Entire Abdominal Cavity at SLL by Presence/Absence of Residual Material – Treated Subjects, Efficacy Population (N=33)**

| **Efficacy Outcome** | **Comparison of all 4 treatment groups** | **Regression analysis models** | |
| --- | --- | --- | --- |
|  |  | **present/**  **not present** | **seen/**  **not seen** |
| Adhesion incidence, n (%) | 0.944 ^a^ | 0.729 ^c^ | 0.925 ^c^ |
| Maximum severity, mean ± SD | 0.567 ^b^ | 0.701 ^d^ | 0.883 ^d^ |
| Mean extent, mean ± SD | 0.342 ^b^ | 0.422 ^d^ | 0.243 ^d^ |
| Adhesion Score, mean ± SD | 0.402 ^b^ | 0.639 ^d^ | 0.938 ^d^ |

^a^ Fisher’s Exact Test p-value;

^b^ Kruskal-Wallis test;

^c^ logistic regression model with residue present/absent and residue seen/not seen as binary predictors;

^d^ linear regression model with residue present/absent and residue seen/not seen as binary predictors
