# Supplementary material for: A first-in-human, randomized, controlled, subject- and reviewer-blinded multicenter study of Actamax™ Adhesion Barrier
Source: Arch Gynecol Obstet. 2016 Nov 14;295(2):383–95. doi: 10.1007/s00404-016-4211-x (PMC5281664; doi:10.1007/s00404-016-4211-x)
Supplement: Supplementary file 5 [file 404_2016_4211_MOESM1_ESM.docx]

Supplemental Online Figure 1. Assessment of Hydrogel Application in all 47 Subjects in whom it was applied – Overall Satisfaction, Viewing, Handling and 12 Aspects of Functionality

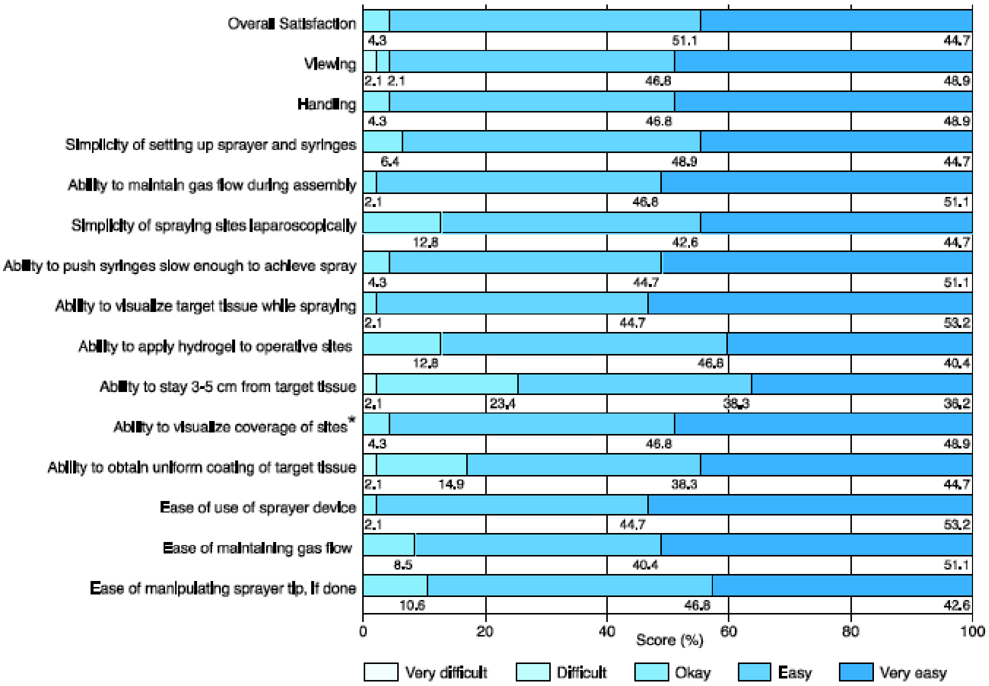

*e.g., how thick the layer was and the quality of the visualization
